# GelMA loaded with platelet lysate promotes skin regeneration and angiogenesis in pressure ulcers by activating STAT3

**DOI:** 10.1038/s41598-024-67304-2

**Published:** 2024-08-07

**Authors:** Tingting Jin, Zexin Fu, Liuyi Zhou, Lulu Chen, Ji Wang, Lu Wang, Sheng Yan, Ting Li, Peihong Jin

**Affiliations:** 1 Center for Plastic and Reconstructive Surgery, Department of Plastic and Reconstructive Surgery, Zhejiang Provincial People’s Hospital (Affiliated People’s Hospital, Hangzhou Medical College), Hangzhou, China; 2 The Second Clinical Medical College, Zhejiang Chinese Medical University, Hangzhou, China

**Keywords:** Pressure ulcers, Platelet lysate, Gelatin methacrylate, Wound healing, Skin, Cell growth, Cell migration, Medical research

## Abstract

Pressure ulcers (PU) are caused by persistent long-term pressure, which compromises the integrity of the epidermis, dermis, and subcutaneous adipose tissue layer by layer, making it difficult to heal. Platelet products such as platelet lysate (PL) can promote tissue regeneration by secreting numerous growth factors based on clinical studies on skin wound healing. However, the components of PL are difficult to retain in wounds. Gelatin methacrylate (GelMA) is a photopolymerizable hydrogel that has lately emerged as a promising material for tissue engineering and regenerative medicine. The PL liquid was extracted, flow cytometrically detected for CD41a markers, and evenly dispersed in the GelMA hydrogel to produce a surplus growth factor hydrogel system (PL@GM). The microstructure of the hydrogel system was observed under a scanning electron microscope, and its sustained release efficiency and biological safety were tested in vitro. Cell viability and migration of human dermal fibroblasts, and tube formation assays of human umbilical vein endothelial cells were applied to evaluate the ability of PL to promote wound healing and regeneration in vitro. Real-time polymerase chain reaction (PCR) and western blot analyses were performed to elucidate the skin regeneration mechanism of PL. We verified PL’s therapeutic effectiveness and histological analysis on the PU model. PL promoted cell viability, migration, wound healing and angiogenesis in vitro. Real-time PCR and western blot indicated PL suppressed inflammation and promoted collagen I synthesis by activating STAT3. PL@GM hydrogel system demonstrated optimal biocompatibility and favorable effects on essential cells for wound healing. PL@GM also significantly stimulated PU healing, skin regeneration, and the formation of subcutaneous collagen and blood vessels. PL@GM could accelerate PU healing by promoting fibroblasts to migrate and secrete collagen and endothelial cells to vascularize. PL@GM promises to be an effective and convenient treatment modality for PU, like chronic wound treatment.

## Introduction

Pressure ulcers (PU) are a worldwide significant problem caused by persistent long-term pressure on specific body tissues through skin ischemia, hypoxia, and ischemia–reperfusion injury^1^. According to epidemiological data, PU affects millions of adults each year, and it will be too costly to prevent all PU^2,3^. The integrity of the epidermis, dermis, subcutaneous adipose tissue, muscle, and bone is gradually undermined layer by layer during the development of PU^1^. The typical pathological features of PU include local tissue ischemia, collagen fiber rupture, and irreversible enlargement and deepening of the wound with the intensification of local inflammation and tissue necrosis^4^. PU causes significant changes in the dermis layer, which contains collagen fibers, elastic fibers, vital blood vessels, and extracellular matrix (ECM). Human dermal fibroblasts (HDFs) are the main cell types in the dermis layer responsible for synthesizing collagen and elastic fibers, playing an important role in wound healing^5,6^. The current standard of treatments for PU relies on flap reconstruction, traditional surgical debridement, negative pressure wound therapy, hyperbaric oxygen, bioengineered skin substitutes, advanced bioactive wound dressings and so on^7–9^. Due to the complex wound pathophysiology of PU, the effects of the aforementioned therapeutic techniques are limited. For example, the skin flaps used for PU are difficult to obtain and survive, and topical agents do not typically provide enough strength or bulk to cover the wound^4^. Therefore, it is necessary to create innovative approaches with proven efficacy for treating PU.

Recently, cell therapy-based techniques have received more and more attention in the field of wound healing. Platelet-derived growth factor has been approved by the US Food and Drug Administration for the treatment of diabetic foot ulcers^10^. Besides, it has been reported to be effective and well tolerated in the treatment of PUI^10^. Platelet-rich plasma (PRP) is one of the most widely used agents that can significantly improve wound healing in chronic diabetic ulcers, venous ulcers, acute traumatic wounds, and ulcers of multifactorial etiologies^11^. However, the preparation technique of PRP lacks standard preparation and cannot be stored for a long time, resulting in unsatisfactory efficacy in clinical settings^12^. As our previous studies reported, platelet lysate (PL) is the growth factor-rich product released by platelets, containing epidermal growth factor (EGF), platelet-derived growth factor (PDGF), insulin-like growth factor-1 (IGF-1) and transforming growth factor-β (TGF-β) by freeze-thawing preparation from platelet concentrates^13–15^. PL is the cell-free product containg high concentrations of growth factors, can be stored at low temperatures for the long time, the use of foreign materials is avoided in the PL preparation process, and can easily be quality controlled for standardization, which are better than other platelet concentrates such as PRP^15,16^. PL has been reported exerting regenerative effects on various tissues, such as skin, cartilage and tendon^17,18^. Recently, our studies demonstrated that PL exerted positive effects on skin by increasing dermal thickness and collagen fibers of nude mice, and also affected HDFs by inducing proliferation and migration, as well as suppressing senescence^14^. Moreover, we found that the growth factors (PDGF, IGF-1, TGF-β and EGF) contributed to the regenerative effects of PL to varying degrees^14^. Therefore, PL may have immense potential for treating PU and benefiting on the process of wound healing.

Although numerous studies have been conducted on using platelet-derived products in the wound treatment, the limitations still exist such as low usage and limited external action time^19^. Biomedical dressings with biomolecular controlled release that can effectively deliver drugs/cells have been extensively studied in wound healing therapy and skin regeneration, which are promising materials for PU^20,21^. In the field of tissue engineering and regenerative medicine, gelatin methacrylate (GelMA) as a photopolymerizable hydrogel, has been extensively researched^22^. GelMA is the highly biocompatible cellular tissue scaffold with optimal temperature-sensitive gel properties, degradability, adjustable mechanical properties, and low antigenicity^23,24^. GelMA can provide a range of viscoelastic properties, facilitate the dispersion of nutrients and oxygen, and provide three-dimensional (3D) support for cells^24^. Consequently, GelMA has been extensively studied for skin regeneration, as it can completely cover the lesion, prevent subsequent infection, and deliver therapeutic molecules^25,26^.

In this study, we aimed to examine the effects of GelMA loaded with PL on the wound healing of PU. We prepared the PL@GM hydrogel by combining PL with GelMA and applying it as a wound dressing to the skin wounds of mice. The effects of PL@GM hydrogel on wound healing, angiogenesis and collagen I (Col-1) were evaluated. The HDFs and human umbilical vein endothelial cells (HUVECs) were treated with PL, and cell viability and migration of HDFs, as well as tube formation of HUVECs, were evaluated to observe the efficacy of PL and PL@GM hydrogel. This study determined the efficacy and mechanism of PL@GM hydrogel on PU for the first time, providing a promising therapeutic modality for wound healing.

## Results

### Characterization of PL and PL@GM hydrogels

**Figure 1:**
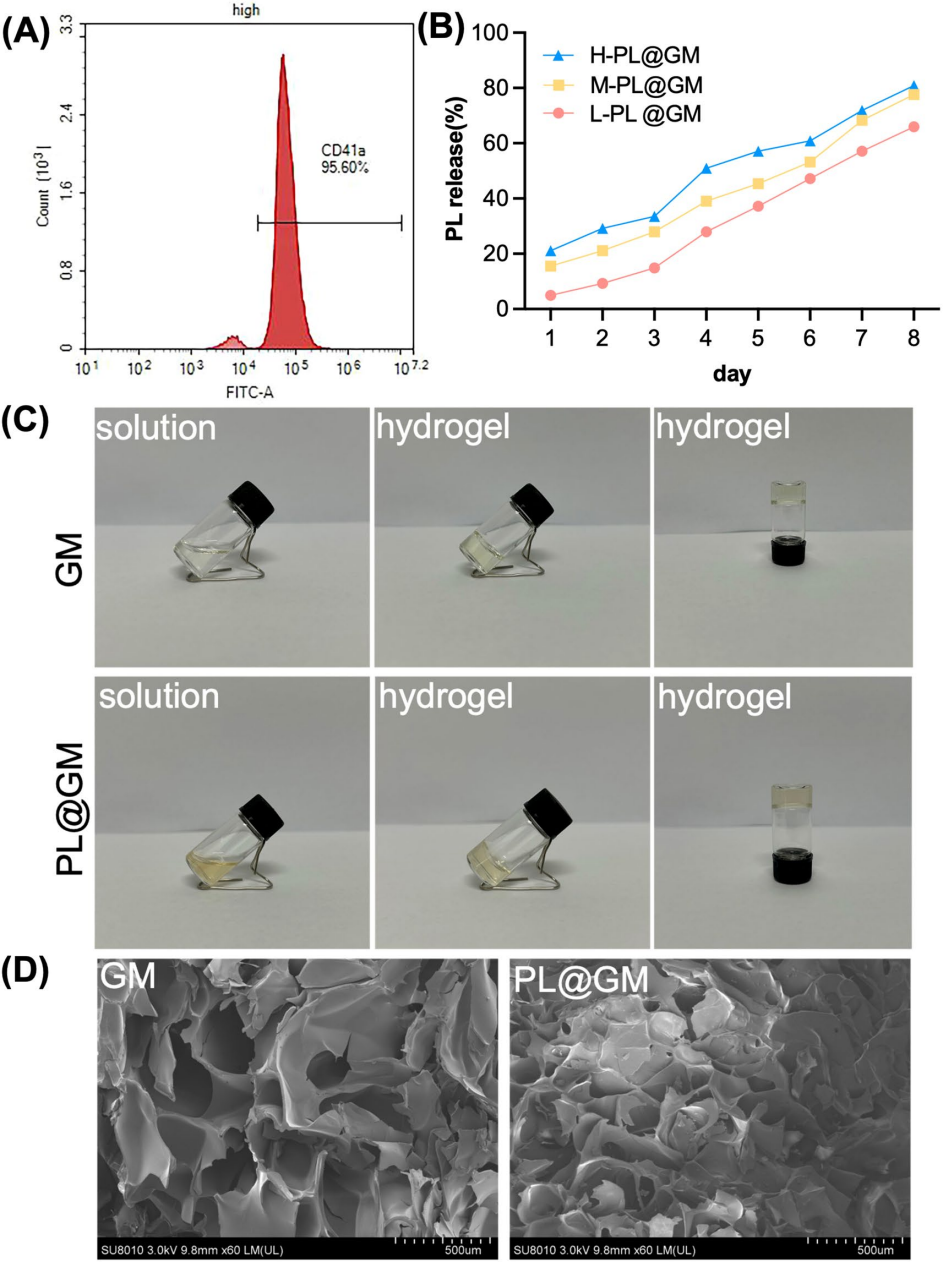
Characterization of PL, GM and PL@GM hydrogel. (**A**) Quantitative CD41a expression on PL via flow cytometry. (**B**) Release efficiency of PL from PL@GM hydrogel system**. (**C) Photographs of the GelMA solution, PL@GM solution, GelMA and PL@GM cross‐linked hydrogels (**D**) SEM images of GM and PL@GM.

As shown in Fig. 1A, flow cytometrical analyses showed more than 95.60% positive expression of CD41a in PL production before freeze–thaw lysis, a specific surface marker for human platelets. The prepared PL was mixed into GM. As shown in Fig. 1B, a protein release profile was observed. In the three concentrations of PL@GM hydrogels, the burst release duration of PL was from day 3 to day 6. The three concentrations of PL@GM hydrogels all steadily and continuously released PL in the hydrogel system. The formation process of GM and PL@GM hydrogels are shown in Fig. 1C. The GM solution is a clear and clarified solution, and PL@GM is a pale yellow, clarified solution with strong flow properties. Subsequent photocuring under 405 nm light provided GM and PL@GM hydrogels in approximately 10 s at 37 °C. After photo-crosslinked and lyophilization, the porous structures were observed by SEM. As shown in Fig. 1D, the PL microparticles was mixed into in the internal structure of GM. It could be observed that hydrogel networks of PL@GM are more compact and have smaller pores, which may improving the mechanical properties of the hydrogels.

### PL promotes HDFs cell viability, wound healing in vitro and angiogenesis

**Figure 2:**
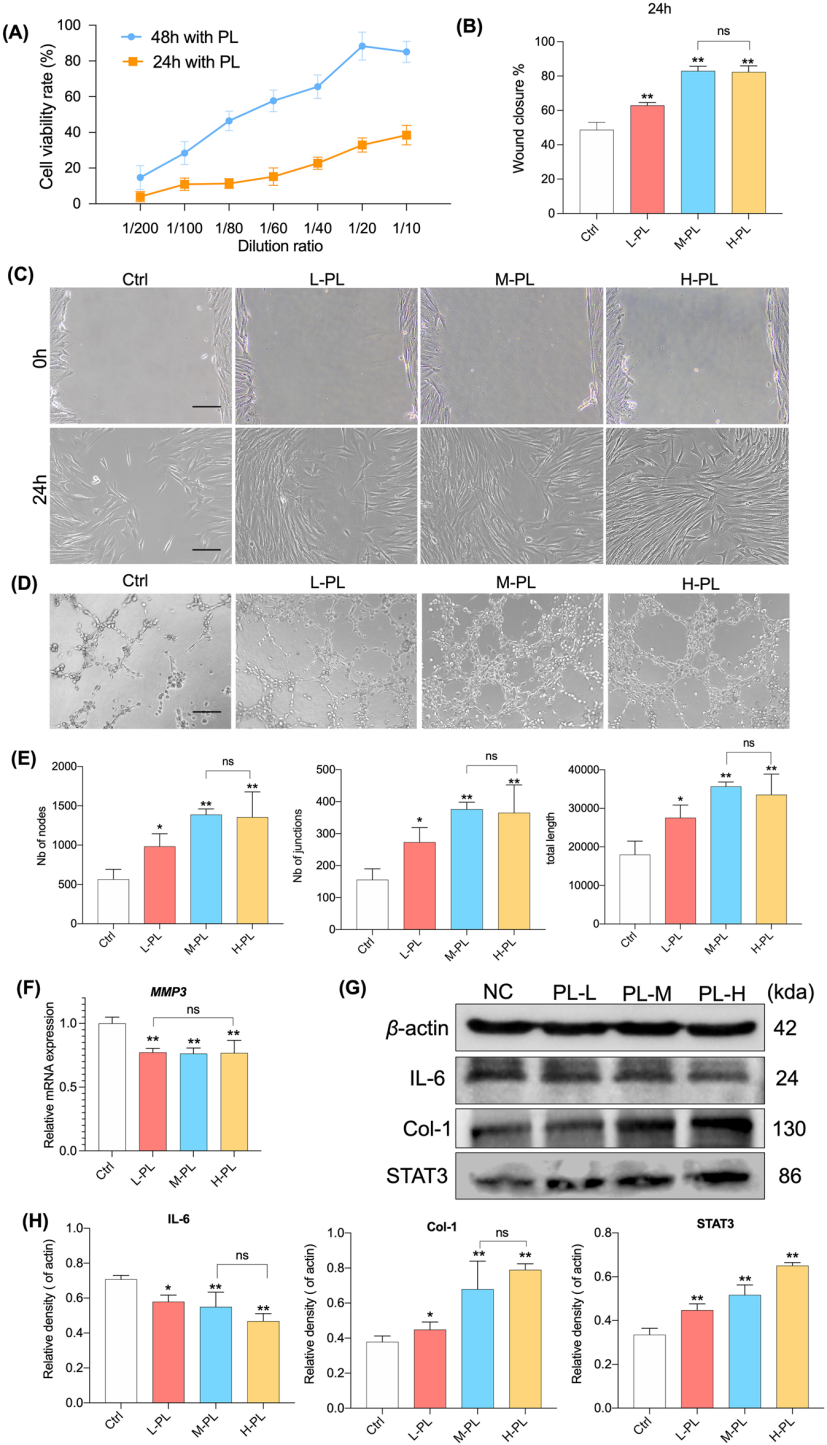
PL stimulates beneficial healing characteristics of HDFs and HUVECs. (**A**) CCK8 assay shows HDFs viability at 24 h and 48 h after PL treatment. (**B**) Cell migration is represented as the ratio of the scratched wound area of HDFs with 24 h PL treatment to the area without treatment (0 h) and (**C**) typical photographs of wound healing assay of HDFs with different concentration gradient PL treatment for 24 h. (**D**) Tube networks formation of HUVECs in the control and different PL groups (scale bar = 200 μm, **C**–**D**). (**E**) The tube formation ability of HUVECs was improved significantly. (**F**) The relative mRNA expression of genes of HDFs with different PL treatments via qPCR. (**G**) and (**H**) Protein bands and their protein expression in HDFs with different PL treatments via WB analysis. All the above data are expressed as mean ± SD.*p < 0.05 or **p < 0.01 vs. control group by one-way ANOVA followed by LSD multiple comparison. We repeated the experiments three times to ensure the accuracy of the experiments.

In this section, we primarily verified the in vitro advantages of PL on chronic wounds. Through CCK8 assays, HDFs viability were evaluated at different dilutions of PL. PL at the dilution rate ranging from 1/200 to 1/20 exerted a significantly viability effect on HDFs in a dose-dependent manner after 24 and 48 h treatment (Fig. 2A). Therefore, we also determined the dilution of 20, 40 and 60-fold of PL represented low, medium and high concentrations for subsequent experiments. Secondly, the effect of different doses of PL on HDFs by promoting migration was evaluated. The percent wound closure in the PL groups was significantly higher than that in the control group (all *p* < 0.01) (Fig. 2B and C). The effect of PL was dose-dependent, while there was no statistically significant difference between the M-PL and H-PL groups (*p* > 0.05) (Fig. 2B and C). Matrigel tube formation assay was applied to evaluate the effect of PL on the tube formation ability of HUVECs. Compared to the control group, with the treatment of PL, the statistical analysis of tube networks on number of nodes (Nb of nodes), number of junctions (Nb of junctions), and total tubule length of tube networks all significantly increased (*p* < 0.05 for each). The M-PL group promoted vasculogenesis more effectively than the H-PL group, although there was no statistical difference between the two groups (Fig. 2D and E). Data from the above study showed that PL induced cell viability and migration of HDFs and tubule network formation in HUVECs.

### PL suppresses inflammation and promotes collagen I synthesis by activating STAT3

qPCR and WB analyses were conducted to elucidate the molecular actions of PL on HDFs. The mRNA expression of *MMP3* were significantly down-regulated with PL treatments (*p* < 0.01 vs. control), while there was no statistical difference among the three PL groups (*p* > 0.05) (Fig. 2F). Furthermore, relative to the control group, the protein expression of Col-1 and STAT3 were significantly up-regulated in PL groups, but those of IL-6 were significantly down-regulated (all *p* < 0.01) (Fig. 2G and H). The expression of these crucial proteins was regulated by PL in a dose-dependent manner. There was no statistical difference between the M-PL and H-PL groups in the expression of IL-6 and Col-1 (*p* > 0.05). The above results indicated that PL inhibited inflammatory factor IL-6 and promoted the expression of Col-1, which could be related to the activation of STAT3.

### In vitro effects of PL@GM hydrogel on HDFs

**Figure 3:**
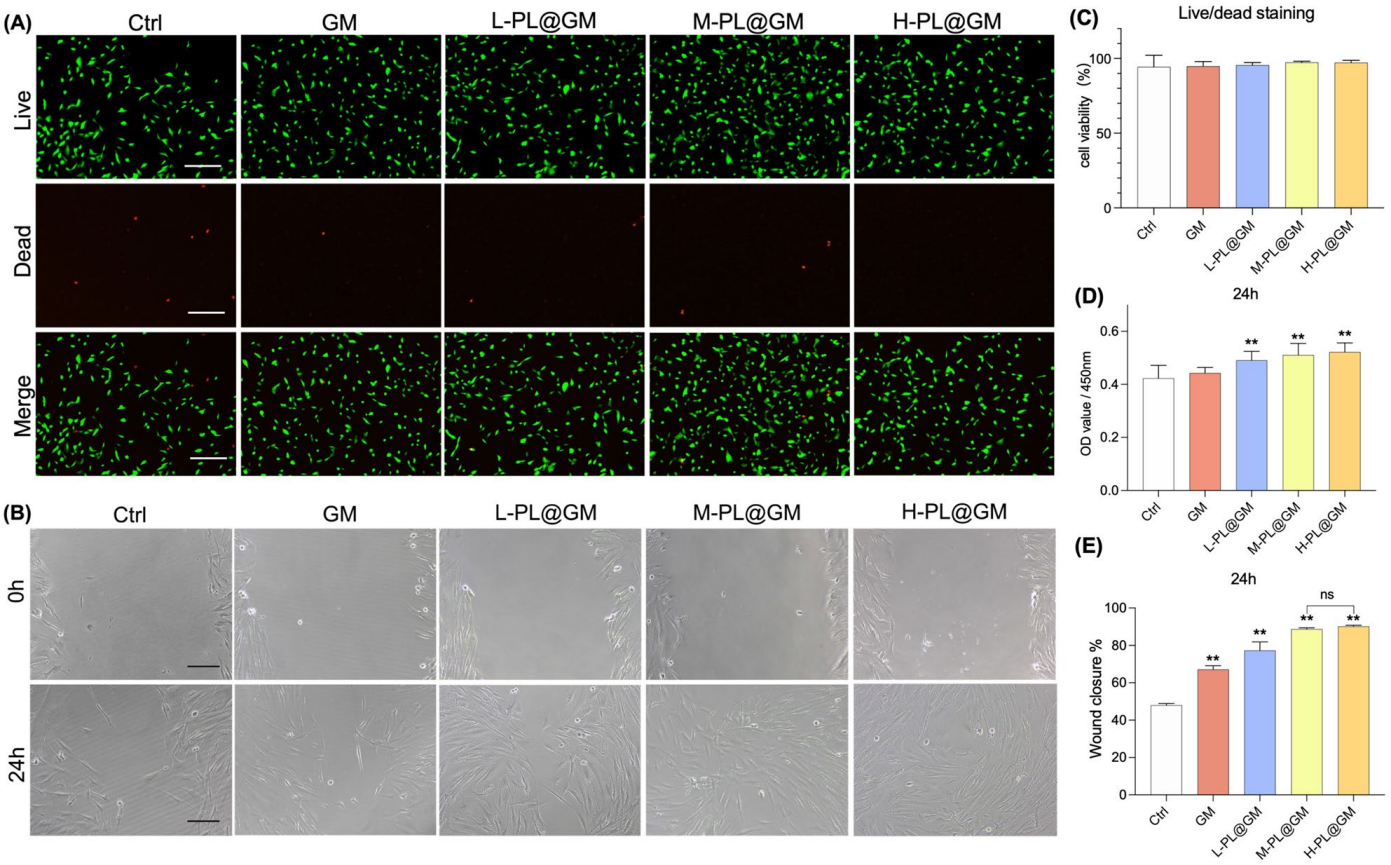
The PL@GM hydrogel can promote the viability and migration of HDFs. (**A**) Live and dead stain assay for GM and PL@GM hydrogel. Scale bar = 200 μm. (**B**) Wound healing assay of HDFs with different concentration gradient PL treatment for 24 h. Scale bar = 200 μm. (**C**) Quantitative analysis for live and dead stains and cell viability is represented as the total ratio of live cells to live and dead cells. (**D**) CCK8 assay shows HDFs viability under the treatment of PL@GM for 24 h. (**E**) Quantitative analysis for wound healing assay and HDFs migration is represented as the ratio of the scratched wound area of HDFs with PL@GM treatment (24 h) to the area without treatment. All the above data are expressed as mean ± SD. *p < 0.05 or **p < 0.01 vs. Control group by one-way ANOVA followed by LSD multiple comparison. We repeated the experiments three times to ensure the accuracy of the experiments.

The biocompatibility and in vitro effects of PL@GM hydrogel were investigated. Live/dead cell staining showed that HDFs plated on the surface of GM and PL@GM hydrogels maintained high activity (Fig. 3A and C). The CCK‐8 assay showed that the viability of HDFs co‐cultured with GM and PL@GM hydrogels. Compared to the control group, L-PL@GM, M-PL@GM and H-PL@GM groups significantly increased the cell viability (all *p* < 0.01), and M-PL@GM displayed the best viability effect on HDFs (Fig. 3D). Moreover, the characteristics of PL@GM in promoting cell migration in the scratch experiment was also confirmed (Fig. 3B and E). With the increase of the proportion of PL in the hydrogel, the level of promoting migration also increased notably, while there was no statistical difference between M-PL and H-PL @GM groups (Fig. 3B and E).

### PL@GM accelerates chronic pressure ulcer wound healing in mice

**Figure 4:**
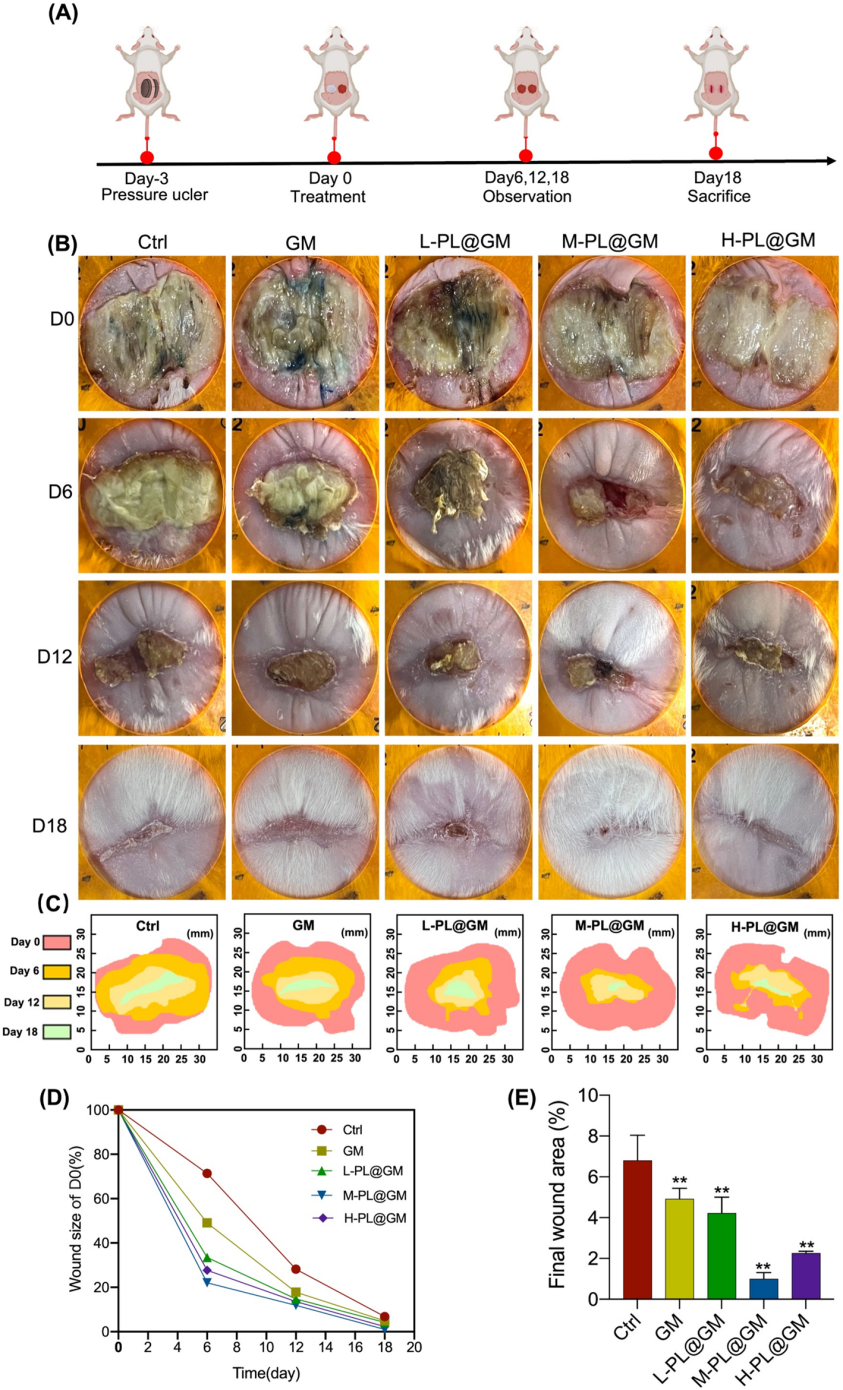
In vivo macro evaluation of wound healing of skin defects in PU animal model. (**A**) Schema for in vivo experiment of PU model and subsequent treatments by PL@GM. (**B**) Representative photographs of PU wound area in each group after treatments. The orange circle is 3 cm in size. (**C**) Simulation diagram and (**D**) quantitative analysis of the change in wound size. (**E**) On day 18, the final wound size of each group after treatments.

We used magnets to repeatedly induce hyperemia, then released them to create a stage III PU mouse model (Fig. 4A). As shown in Fig. 4B and C, from the general photographs and simulated map of wound size changes, it could be seen that the wound healing process was accelerated by GM and each PL@GM. M-PL@GM group healed significantly faster than other groups on day 6, 12 and 18 (Fig. 4B and C). On day 18, the scar in all hydrogel groups was notably smaller than in the PU control group (Fig. 4B). As shown in Fig. 4D and E, the rate of wound size/D0 and the final area of the three concentrations of L-PL@GM, M-PL@GM and H-PL@GM groups all smaller than the control group (all *p* < 0.01). M-PL@GM group exerted the best effect on promoting the wound healing and showed the smallest final wound area, and there was no statistical difference between the M-PL@GM and H-PL@GM group. These results verified that GelMA could benfit on the wound area and deliver PL to promote chronic cutaneous wound healing of PU.

### PL@GM promotes collagen regeneration and angiogenesis in the PU mouse model

**Figure 5:**
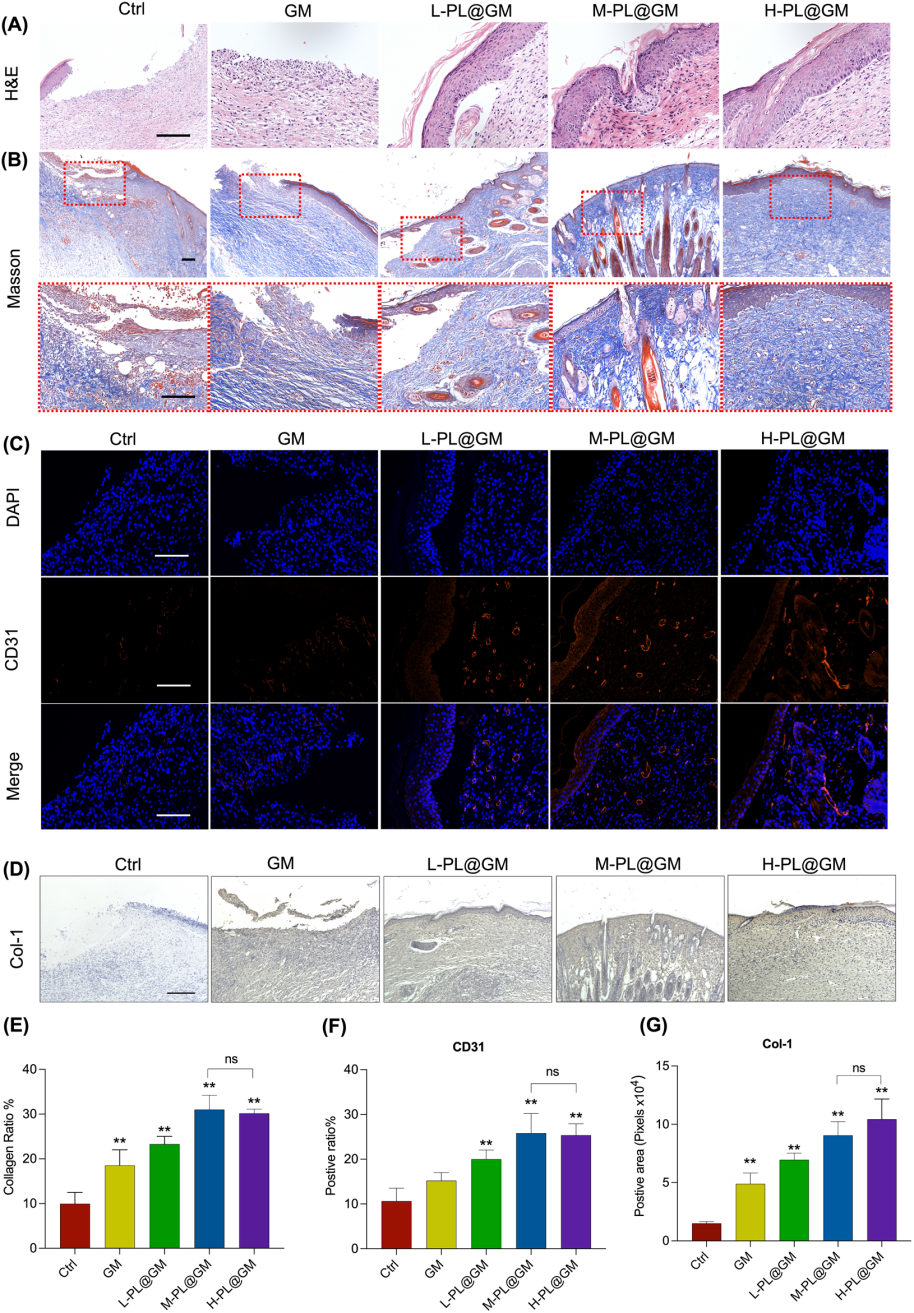
In vivo histopathological observation. (**A**) H&E staining of PU wound site of differernt groups. (**B**) Masson's trichrome staining and magnification images. (**C**) Immunofluorescence analysis of CD31 of the wound site. (**D**) Immunohistochemistry staining images of Col-1 of the wound site. Scale bar = 200 μm. (**E**) Quantitative analysis of collagen ratio. (**F**) Average fluorescence intensity score of CD31. (**G**) Quantitative analysis of Col-1 expression. Data expressed as mean ± SD. *p < 0.05 or **p < 0.01 vs. control group by one-way ANOVA followed by LSD multiple comparison. We repeated the experiments three times to ensure the accuracy of the experiments.

Histological investigation revealed additional evidence that PL@GM accelerates chronic wound healing. The control, GM, and L-PL@GM groups failed to achieve complete wound healing based on H&E staining. Compared with control group, GM and PL@GM groups accelerated wound healing. Wounds treated with PL@GM groups had more granulation and dermal tissue to fill the defect, and the continuous epidermal layer was observed in M-PL@GM and H-PL @GM groups (Fig. 5A). Masson's staining was used to visualize the thickness of collagen fibers. For masson staining, we were particularly concerned about collagen production and order. The collagen fibers were disrupted with a significant decrease in collagen levels in the control group (Fig. 5B). Compared with control group, the abnormalities were remarkably restored and there was a significant increase in the collagen thickness level in the GM and PL@GM groups (*p* < 0.01) (Fig. 5B and E). The M-PL@GM group showed the best results, but the difference between the M-PL@GM and H-PL@GM group was not statistically significant (Fig. 5B and E). Regarding angiogenesis, CD31 immunofluorescence results are shown in Fig. 5C and F. Compared to the control group, the CD31 expression of all PL@GM groups was significantly increased (all *p* < 0.01), and there was no statistical difference between M-PL@GM and H-PL@GM group in the CD31 expression (*p* > 0.05) (Fig. 5C and F). For immunohistochemistry, compared with the control group, the Col-1 expression of all PL@GM groups was significantly increased in a dose-dependent manner (Fig. 5D and G). The above data revealed that PL promotes skin regeneration by restoring dermal and epidermal structure, increasing collagen fiber ratio and promoting the expression of Col-1 and CD31.

## Discussion

Our previous studies have reported that PL contained rich growth factors like TGF-β, IGF-1, EGF and FGF, possessing a pro-regenerative effect on skin^14^, which may have outstanding performance in repairing chronic wounds. Other studies indicate that platelet-related derivatives contribute to angiogenesis in *in-vitro* 3D endothelial bioprinted constructs^27^, dental pulp^28^, and bone^28^. Considering the pathophysiological mechanism of PU, we assume that a stable supply of PL benefits the wound healing and subcutaneous tissue of PU. To determine this, our study devised a drug delivery system consisting of PL loaded with GelMA hydrogel.

Firstly, in vivo, we evidenced the angiogenesis, collagen formation and wound healing of PL@GM against chronic wounds through animal experiments. As reported in the previous study, SEM images of PL formulated with GelMA showed uniform surface morphology, with spherical structures (proteins fromque PL) adhering to the polymer chains at very high magnification^29^. In addition, PL formulated with GelMA hydrogels reduced the overall water absorption of the network, which showed the smaller pores and a dense network structure^30^. PL-GelMA hydrogels release proteins, support cell viability and differentiation, and improve the mechanical properties of hydrogels^29,31^. PL@GM showed a pro-regenerative effect and accelerated wound healing by dose-dependently increasing subcutaneous vascularization (CD31 expression) and dermal thickness, promoting collagen remodeling and follicle growth in mice. In the gross photograph of the final outcome, the wound area of the M-PL@GM group was significantly smaller than that of other groups, and new hair was formed. The wound size of the GM group was also smaller than that of the control group, which could be attributed to moist healing as it is more favorable to chronic wound recovery and reduced scar formation^32^. There are stages of inflammation, tissue development, angiogenesis, tissue contraction, and tissue remodeling during the wound healing process. Cellular interactions with the extracellular matrix (ECM) are critical for these events^6,32^. In the skin dermal ECM, Col-1 is the main component accounting for the total collagen fibers which arranged as a macromolecule in the ECM, providing tensile strength and elasticity to promote wound healing^33,34^. CD31 is a vascular marker, which is important for characterizing the vessel in the skin. In the aspect of skin, the vessels contribute to maintaining dermal and epidermis homeostasis. Notably, angiogenesis is the important component in promoting wound healing when the skin is confronted with injury^35–37^.

In addition to epidermal cells, HDFs also play a significant role in skin wound healing. The rapid proliferation and migration of HDFs after injury determines the contraction and closure of the wound bed and the integrity of the newly formed dermis^38^. In vitro, PL exerted a positive effect on HDFs by improving cell viability and migration, inhibiting expression of inflammatory and degrading molecules (IL-6 and *MMP3*) and altering ECM-related markers of HDFs (Col-1 and STAT3) (Fig. 2). MMPs are secreted by crucial skin cells (such as HDFs) and deteriorate ECM environment^39^. MMP-3 acts as a stromelysin, which may contribute to the degradation of fibrillar collagen, collagen type I/II, and elastin fibrils of skin, finally delaying wound healing^40^. The cytokines IL-6, the hallmark of inflammation, reinforces the growth arrest and increase in wound tissue^41^. Our results showed that PL had significant angiogenic capacity by promoting HUVECs to tubule network formation. Angiogenesis is particularly crucial to the proliferation phase of wound healing, while endothelial cells are responsible for forming new vessels^42,43^. STAT3 is a member of the STAT (signal transduction and transcriptional activator) family. Many cytokines and growth factors can activate STAT3, which is important in cell activity, oxidative stress response, metabolism and autophagy^44^. In wound healing progress, STAT3 was reported to directly induce the expression of VEGF to promote angiogenesis^45^. Another study has also reported that activating STAT3 signaling and downstream effectors in the skin can promote epidermal proliferation and wound reepithelialization^46^. In this study, the protein expression of Col-1, IL-6 and STAT3 were reversed by PL treatment, indicating STAT3 may mediate its molecular mechanism of paracrine action (Fig. 2), warranting further investigations.

**Figure 6:**
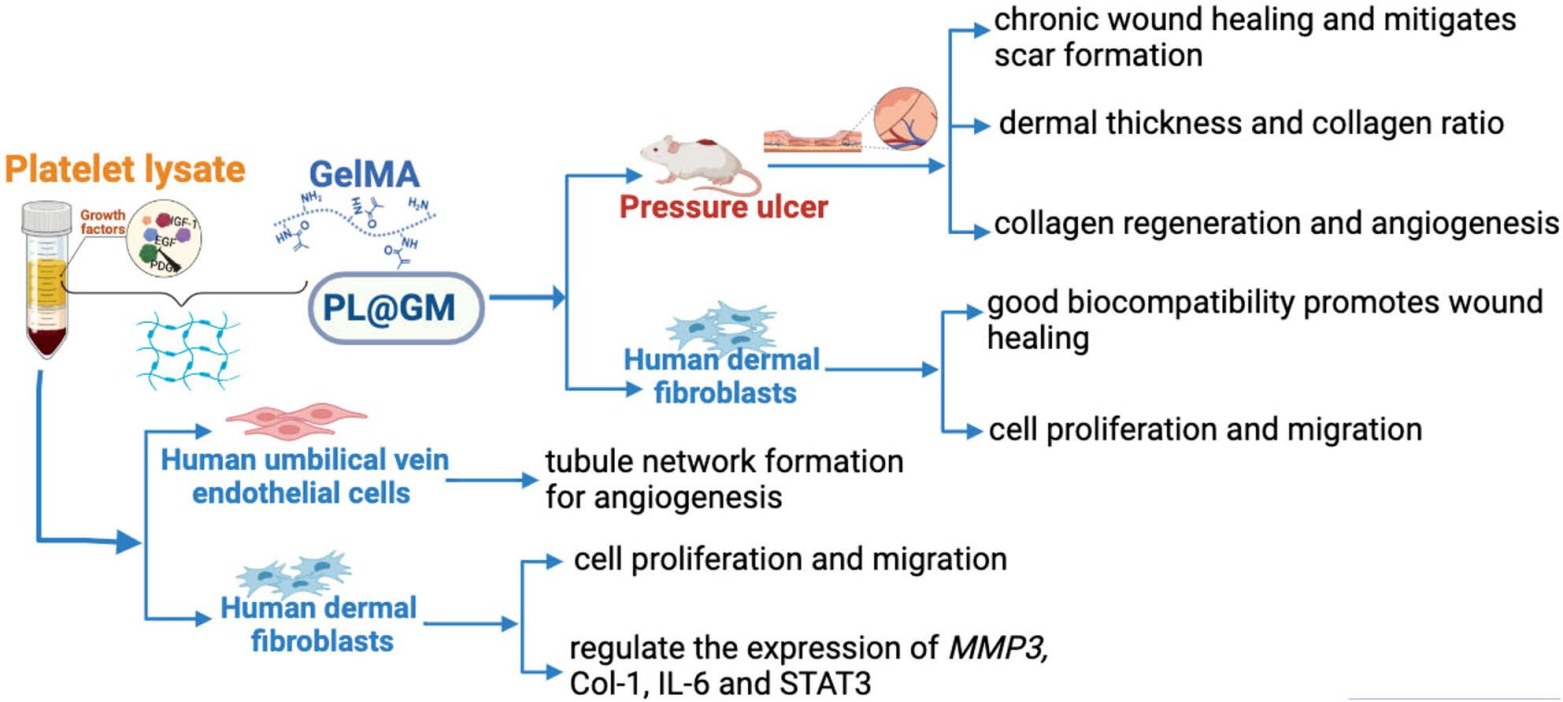
Schematic diagram of the efficacy of PL and PL@GM hydrogel on PU.

In our study, from all of the above results, we demonstrated that PL can continuously be released in the GelMA system, and PL@GM hydrogel has a potent effect in promoting the healing process of PU and regeneration process (Figs. 4 and 5). The PL@GM hydrogel also showed excellent cytocompatibility. It can significantly accelerate the viability and migration of HDFs (Figs. 1 and 3). GelMA contains arginine-glycine-aspartic acid (RGD), which facilitates the attachment, spreading and differentiation of certain cells into a variety of lineages that play a key role in skin wound healing, morphogenesis and restoration^22^. In recent years, GelMA has been utilized in numerous areas of skin regeneration as the representative hydrogel formulation, which can be loaded with various extracellular vesicles or human mesenchymal stem cell-conditioned media to stimulate skin regeneration^26,47^. GelMA has been demonstrated to provide the environment for HUVEC to form monolayers of cells and foster angiogenesis^48^. Our previous study reported that the growth factors in PL may synergistically contribute to the positive effects of PL on skin^14^. ECM remodeling and angiogenesis are important for chronic wounds such as PU^49^. For the first time we report evidence that PL functions through the growth factor-based pro-regenerative mechanism, and that the GelMA hydrogel system can provide a PL-sustained release therapy (Fig. 6). Therefore, GelMA loaded with PL may be an ideal therapeutic modality for wound healing of PU.

## Conclusion

This study found that GelMA loaded with PL (PL@GM) could accelerate wound healing by promoting angiogenesis and skin ECM regeneration. GelMA loaded with PL also exerted beneficial effects on HDFs by cell viability and migration. PL is beneficial to the essential cells in the process of wound healing, promoting fibroblasts to migrate and secrete collagen. PL can also stimulate endothelial cells to vascularize.

The mechanism may be related to STAT3, warranting further investigations. In the PL@GM hydrogel system, slow and long-term release of PL can aid the healing of chronic pressure sores. The results of this study support the notion that hydrogel sustained-release PL systems can be an effective, cost-effective, and convenient method of treating chronic wounds like PU.

## Materials and methods

### Chemicals and reagents

HDFs were provided by Kunming Cell Bank of Chinese Academy of Sciences (KCB200537). HUVECs were purchased from the Chinese Academy of Sciences (Beijing, China). High dulbecco’s modified Eagle medium (H-DMEM), fetal bovine serum (FBS), 100X Penicillin–Streptomycin Solution, phosphate-buffered saline (PBS), trypsin and Cell Counting Kit-8 (CCK-8) were sourced from Dalian Meilun Biotechnology Co., Ltd. (Dalian, P. R. China). The miScript SYBR Green and PCR kit was obtained from Qiagen (Dusseldorf, Germany). BCA Protein Assay Kit was purchased from Beyotime Institute of Biotechnology (JiangSu, China). The antibodies human platelet surface marker CD41a, collagen type I (Col-1), Signal Transducer And Activator Of Transcription 3(STAT3), β-actin and Interleukin- 6 (IL-6) were purchased from Proteintech Group, Inc (Wuhan, China). HRP-labeled goat anti-mouse antibodies was bought from Gene Tech (Shanghai, China). GelMA-90 (DS: 90%) and ithium phenyl-2, 4, 6-trimethylbenzoylphosphinate (LAP) were bought from Yongqinquan Intelligent Equipment Co., Ltd. (Suzhou, China). The Matrigel (10 mg/ml; #354230) was sourced from Corning (New York, USA).

### PL preparation

With the informed consent of one healthy adult blood donor, the whole blood was collected in an anticoagulant tube. The whole blood was centrifuged at 210 g for 10 min, and the yellow plasma of ESR was collected in a new tube and centrifuged again at 210 g for 5 min. The purified platelet concentrate was acquired. The residual red blood cells were removed, and the platelets were fully lysed by repeated circulation of freezing and thawing. The final PL was obtained. To determine the purity of platelet concentration, the human platelet surface marker (CD41a) was analyzed via flow cytometry.

### Fabrication of GelMA hydrogels and PL@GM hydrogels

10% GelMA-90 (w/v) (DS:90%) hydrogels were prepared by dissolving the GelMA sponge in prepared PBS with 0.05% phenyl-2,4,6-trimethyl-benzoyl phosphate lithium (LAP) at 60 °C for 30 min and stirred every 10 min.

To fabricate PL@GM, GelMA-90 (DS: 90%) was resuspendended at 10% (w/v) PBS with the 0.05% photoinitiator lithium phenyl-2, 4, 6-trimethylbenzoylphosphinate (LAP). And PL was diluted 20, 40, or 60 fold in 10% GM-90, representing the H-PL@GM, M-PL@GM and L-PL@GM respectively. Exposed by 405 nm blue light, PL@GM was quickly cross-linked to form the hydrogel for 10–20 s.

### Cell viability assay

HDFs were cultured in high‐DMEM supplemented with 10% FBS and maintained at 37 °C with 5% CO_2_. The cell viability of HDFs was evaluated by CCK-8 assay at 24 and 48 h. Cells were plated on 96-well plates at a density of 5 × 10^3^ cells/well in 150 μl medium. After 24 h, HDFs were treated with dilution rates of 1/20–1/100 gradient concentrations of PL. 10% CCK-8 solution was added to each well and incubated at 37 °C for 1.5 h. The optical density value was measured at 450 nm with a microplate reader (Bio-Rad Laboratories, Inc., USA). Each experiment was conducted in triplicate.

### Scanning electron microscopy (SEM) analysis of GelMA and PL@GM hydrogels

The GelMA and PL@GM hydrogels were prepared as previous method described. Exposed by 405 nm blue light, The 10% GelMA and PL @GM were quickly cross-linked to form the hydrogel. Then the samples were fully freeze-dried and were coated with gold–palladium, which observed under a scanning electron microscope (Nova Nano 450, USA).

### Measurement of PL release in PL@GM hydrogel

To measure the release effect of PL from PL@GM hydrogels, the hydrogels were placed in 1 ml PBS (pH = 7.4) in an incubator at 37 °C. The amount of released PL was detected every 24 h for 8 days. After incubation for 24 h at each time point, 10 μL of each released buffer was taken out, 10 μl of corresponding fresh buffer and 200 μL of Bicinchoninic Acid reagent (Reagent A: Reagent B = 50:1) was added. After incubation for 20 min, the optical density value of the supernatant was measured at 562 nm. The released PL was quantified using Enhanced BCA Protein Assay Kit (Beyotime, Shanghai, China).

### Biocompatibility of PL@GM hydrogel

We used the live/dead staining and CCK8 method to assess the biocompatibility of PL@GM. Hydrogels and hydrogels loaded with L-PL, M-PL and H-PL were put on the rim of the 6-well plate. HDF cells were seeded in the plate and cultured at 37 °C for 24 h. After the supernatant was aspirated and hydrogels were removed, 1 mL Calcein AM/PI reagent (Calcein AM: PI : PBS = 1 μl: 1 μl: 1 ml, Beyotime, Shanghai, China) was added to each well of the plate. After incubation for 30 min, the pictures of cell staining were shot by fluorescence microscopy (EVOS M7000, Thermo Fisher, USA). the image J was used for fluorescence quantification^50^.

### Wound healing assay

As previously described^14^, HDFs were cultured in 6-well plates (10^5^/well) and artificially formed scratched area, the cell monolayer was scraped in a straight line with a p200 pipette tip to form a "scratch", and the cells were washed once with 1 ml of growth medium to remove debris and smooth the edges of the scratch^51^. Then treatment of PL with serum-free medium. The cells were observed and imaged at two different time points (0, 24 h) under a microscope (EVOS M7000, Thermo Fisher, USA). The scratched area was calculated with Image J software. Percent wound closure = (cell-free space at 0 h − cell-free space at 24 h)/cell-free space at 0 h. Two wound healing assays using HDF were conducted, one tested PL and the other tested PL@GM hydrogel. Each experiment was conducted in triplicate. All groups of PL@GM hydrogel systems were suspended in a medium with a 3D-printing bracket. The remaining settings were described above and wound be observed after 24 h.

### Tube formation assay

The HUVEC cells were starved overnight using a cell culture medium supplemented with 0.2% serum before performing the tube formation assay. The 24-well plates were coated with 300 µl of Matrigel (10 mg/ml) and incubated at 37 °C for 30 min to promote gelation. 5 × 10^4^ HUVEC cells were resuspended in 1 ml medium without serum containing 20, 40 or 60-fold diluted PL. After incubation at 37 °C for 6 h, the tube formation phenomenon was observed by an optical microscope.

### Quantitative real-time polymerase chain reaction (qRT-PCR)

The mRNA expression of targeted genes in HDFs cells were measured using a qPCR assay on an ABI QuantStudio™ 7 Flex Real-Time PCR System (Applied Biosystems; Thermo Scientific, USA). Total RNA was extracted with TRIzol reagent. cDNA reverse transcription was performed by using All-in-One cDNA Synthesis SuperMix. Then, the SYBR® Premix Ex Taq II (Tli RnaseH Plus), forward primer, and reverse primer of target gene were used in the process of cDNA amplification, with the following reaction conditions: initial denaturation at 95 °C for 5 min, 40 cycles of denaturation at 95 °C for 3 s, and annealing and extension at 60 °C C for 30 s. *β*-Actin was used as reference gene and the 2^−Δ∆CT^ method was used to analyze (Table 1). The sequences of forward and reverse primer were presented in Table 1.

**Table 1:** Primer sequences used for qPCR analysis.

Gene	Forward primer	Reverse primer
*β-ACTIN*	5′-CCCGCGAGTACAACCTTCT-3′	5′-CGTCATCCATGGCGAACT-3′
*MMP3*	5′-GAGGCATCCACACCCTAGGTT-3′	5′-TCAGAAATGGCTGCATCGATT-3′

### Western blot

Total cellular proteins of HDFs were extracted with radioimmunoprecipitation assay (RIPA) buffer with proteinase inhibitor cocktail for 30 min on ice. 41.88 µg total protein per lane were loaded. The targeted protein was separated by 8–12% SDS-PAGE and transferred onto a nitrocellulose membrane. The membrane was blocked with 5% nonfat milk for 2 h, which was followed by overnight incubation at 4 °C with the following primary antibodies against *β*-actin, IL-6, Col-1 and STAT3. Following incubation with peroxidase-conjugated goat antirabbit/mouse IgG at 4 °C for 2 h, each protein was visualized using Western Lightning® Plus ECL, visualized by GE ImageQuant LAS4000 System 1 (Bio-Rad, Hercules, CA, USA). Results were analyzed with Image J software (version: 1.6.0). Each experiment was conducted three times. The original data is shown in supplementary figure.

### Animal experiments

This study was carried out in compliance with the ARRIVE (Animal Research: Reporting of In vivo Animal Experiments) guidelines. A total of 30 female 7-week-old BALB/c mice were provided by GemPharmatech Co., Ltd (animal production licence number: SCXK: 2019‐0009, Jiangsu, China). All experiments on the mice were approved by the Laboratory Animal Management and Ethics Committee of Zhejiang Provincial People’s Hospital (Approval number:SYXK: 2019‐0013, Zhejiang, China). All experiments were performed in accordance with relevant guidelines and regulations.

The circular magnetic plates (12 mm diameter, ≈ 50 mmHg pressure) were selected for PU modeling. The whole layer of mouse skin (with subcutaneous muscle tissue) was gently pulled up and placed between two round magnetic plates. Two magnetic plates pressurized the mouse skin for 12 h, then the magnetic plates were released for another 12 h to form a single 24-h ischemia–reperfusion cycle (12 h with magnetic plate + 12 h without magnetic plate)." Every mouse was conducted three ischemia–reperfusion cycles to form a PU model, resulting in a III-phase stress ulcer (full-thickness skin defect, occasionally accompanied by subcutaneous tissue and muscle layer injury). Animals were allowed to move, eat and drink freely during modeling without anesthesia or other treatment. After PU modelling for 3 III-phase stress ulcer, Wounds of mice in GM and PL@GM hydrogels groups were covered subcutaneously with PL at dilution rates of 1/20, 1/40, and 1/60 in 10% GM-90, while control groups received no treatments. All treatments were weekly conducted for 2 weeks. At the end of study (D18), all the mice were sacrificed with an overdose of sodium pentobarbital (200 mg/kg; i.p.) and the pieces of skin from the back were taken for histological and biochemical examination.

### Histological analysis

All mice skin was fixed with 10% formalin for 24 h at room temperature. Then each sample was embedded in paraffin and sectioned into 4 μm, followed by H&E staining and Masson staining. The stained sections were observed under microscopy and analyzed by Image J software^52,53^.

Unstained replicates of the sections were incubated overnight at 4 °C with PBS-diluted primary antibodies against mice CD31 for Immunofluorescence. After PBS wash, the sections were incubated with Horseradish peroxidase (HRP) conjugated secondary antibodies for 1 h at room temperature, followed by DAPI Staining Solution for 5 min. The sections were visualized under a fluorescence microscope (EVOS M7000, Thermo Fisher, USA). The immunoreactivity of CD31 was quantified by Image J software (Media Cybernetics, Bethesda, MD, USA)^53^.

For immunohistochemistry, paraffin sections were blocked at room temperature with 5% goat serum in PBS for 30 min and incubated with anti-mouse Col-1 antibody, followed by HRP-labeled goat anti-mouse antibodies for 30 min at room temperature.

### Statistical analysis

All data were analyzed using IBM SPSS Statistics software (V22, IBM Corporation, USA) and expressed as mean values ± standard deviation. Data from different groups were compared using one-way ANOVA or non-parametric tests. A *p* value < 0.05 was considered as a significant difference, and a *p* value < 0.01 was considered to indicate a very significant difference.

### Supplementary Information

Supplementary Information 1.

## Data Availability

The data that support the findings of this study are available from the corresponding author upon reasonable request.
