# Supplementary figures and images for: GelMA loaded with platelet lysate promotes skin regeneration and angiogenesis in pressure ulcers by activating STAT3
Source: Sci Rep. 2024 Aug 7;14:18345. doi: 10.1038/s41598-024-67304-2 (PMC11306777; doi:10.1038/s41598-024-67304-2)

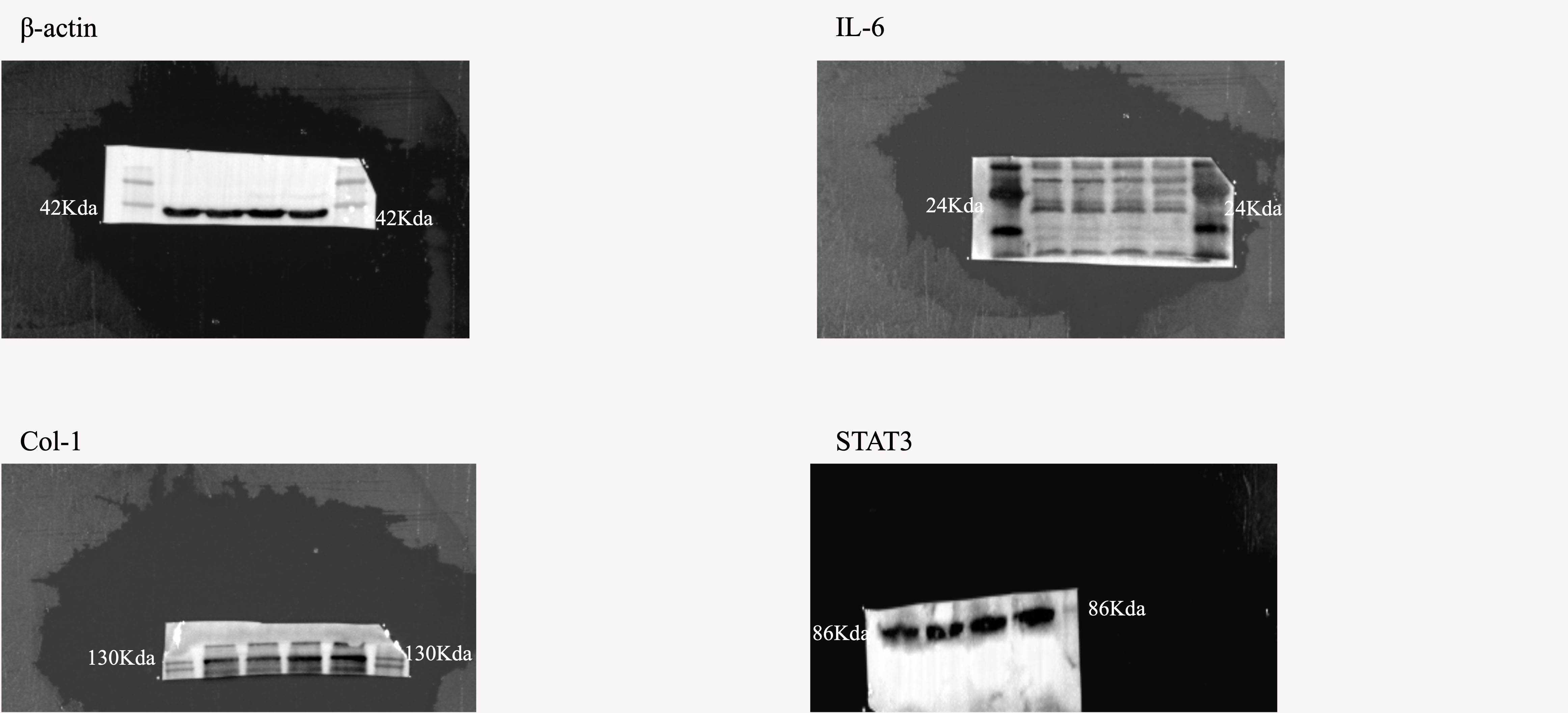

Supplement: Supplementary file 1 — Supplementary Information 1. [file 41598_2024_67304_MOESM1_ESM.jpg]
